# Sealed-Off Uterine Rupture: A Report of a Rare Case

**DOI:** 10.7759/cureus.86769

**Published:** 2025-06-25

**Authors:** Vimal Chaudhary, Naik Deepti, Anil K Sakalecha

**Affiliations:** 1 Radiodiagnosis, Sri Devaraj Urs Academy of Higher Education and Research, Kolar, IND

**Keywords:** emergency obstetric care, fetal demise with uterine rupture, sealed off uterine rupture, second trimester bleeding, spontaneous uterine rupture

## Abstract

Uterine rupture is a rare but potentially life-threatening obstetric complication, most commonly associated with labor in women with a scarred uterus. However, spontaneous rupture in an unscarred uterus, particularly in the second trimester and in primigravida patients, is exceedingly rare and often underrecognized.

We present the case of a 26-year-old primigravida woman at approximately 21 weeks of gestation who presented with acute abdominal pain and vaginal bleeding. Ultrasound revealed a 3.1 cm defect in the uterine fundus, with herniation of fetal parts suggestive of a sealed-off uterine rupture. Fetal demise was confirmed sonographically.

This case highlights the importance of maintaining a high index of suspicion for uterine rupture, even in early gestation and in the absence of prior uterine surgery. Radiological imaging, especially ultrasonography, is pivotal in early diagnosis and timely intervention to minimize maternal risk.

## Introduction

Uterine rupture is a rare but serious obstetric emergency with risks to both mother and fetus. Its incidence is estimated at 0.07% of all pregnancies in developed countries, predominantly occurring in women with prior cesarean delivery or uterine surgery [1]. The majority of reported cases involve laboring women with scarred uteri, particularly in the third trimester, often presenting acutely with abdominal pain, fetal distress, or signs of hypovolemic shock.

Spontaneous rupture of an unscarred uterus is exceedingly rare, with a reported incidence ranging from 1 in 8,000 to 1 in 15,000 pregnancies [2]. Second-trimester rupture in a primigravida is rarely reported. In a 2018 report by Hawkins et al. [3], spontaneous uterine rupture was observed at 21 weeks in a primigravida woman, which was successfully managed with surgical repair and continuation of the pregnancy, highlighting the possibility of early detection and favorable outcomes in select cases.

Proposed causes of rupture in an unscarred uterus include uterine anomalies, connective tissue disorders, placenta percreta, and subclinical infections. In some cases, no identifiable cause is found, making diagnosis even more challenging [4].

Ultrasound plays a central role in the diagnostic approach, especially when clinical signs are nonspecific. Characteristic sonographic findings include a discontinuity in the uterine wall, herniation of amniotic sac or fetal parts into the peritoneal cavity, and signs of fetal demise such as the Spalding sign. However, the “sealed-off” variant of rupture, where the contents remain partially contained within the peritoneal cavity, may present without overt hemodynamic compromise, leading to delayed recognition.

In summary, this case report aims to highlight a rare presentation of spontaneous uterine rupture in an unscarred, early gestational uterus of a primigravida patient without classic risk factors. It underscores the diagnostic challenges and emphasizes the critical role of imaging in the timely identification and management of such atypical but life-threatening obstetric emergencies.

## Case presentation

Patient evaluation

A 26-year-old primigravida presented to the emergency department of Sri Devaraj Urs Medical College on October 17, 2023, with complaints of abdominal pain and vaginal bleeding. She was well-oriented, but appeared restless. Physical exam suggested advanced gestation, confirmed by ultrasound showing fetal biometric measurements corresponding to approximately 21 weeks and 5 days.

An obstetric ultrasonography was performed using a PHILIPS EPIQ 5 ultrasound system (Philips Healthcare, Andover, MA, USA) with a curvilinear broadband transducer (C5-1 MHz). The fetal biometry, uterine wall integrity, placental position, and fetal viability were evaluated. The results were as stated: based on biometric parameters, the estimated gestational age was 21 weeks and 5 days (±2 weeks). Fetal biometry measurements were as follows: the biparietal diameter (BPD) measured 4.67 cm, corresponding to 20 weeks and 1 day; the head circumference (HC) was 18.24 cm, consistent with 20 weeks and 5 days; the abdominal circumference (AC) measured 19.06 cm, correlating with 23 weeks and 6 days; and the femur length (FL) was 3.76 cm, corresponding to 22 weeks and 1 day.

Uterine findings

**Figure 1:**
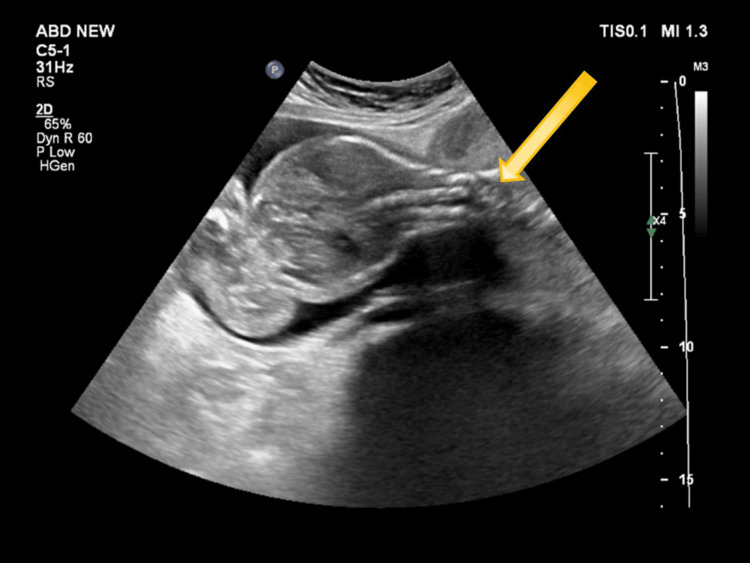
Grayscale ultrasonography image showing a discontinuity in the anterior uterine wall (yellow arrow).

**Figure 2:**
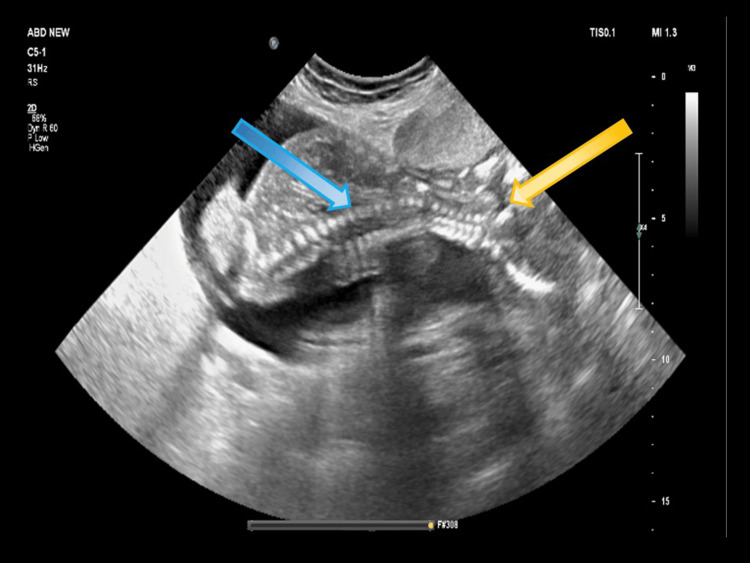
Grayscale ultrasonography image showing uterine rupture (yellow arrow) with spine of the fetus in the peritoneum (blue arrow).

A 3.1 cm defect was noted in the uterine fundus, as demonstrated in Figure 1. There was herniation of the amniotic sac and fetal parts through the ruptured site into the peritoneal cavity, as shown in Figure 2. Fetal parts were visualized adjacent to the liver. No fetal cardiac activity or movements were observed at the time of the scan. The presence of overlapping fetal skull bones (Spalding sign) confirmed intrauterine fetal demise. The placenta was located anteriorly, appeared grade II, and was low-lying, measuring 1.2 cm from the internal os. The cervical length was measured at 3.2 cm.

The patient then underwent emergency laparotomy following stabilization, revealing uterine fundal rupture with herniated fetal parts. The fetus was delivered, confirming intrauterine demise, and the uterine defect was surgically repaired. Postoperative care included IV fluids, blood transfusion, antibiotics, analgesics, and close monitoring. Psychological support was provided for fetal loss. On follow-up, the patient showed stable recovery. She was advised to avoid conception for at least one year and counseled about the high-risk nature of future pregnancies, with a recommendation for early cesarean delivery in subsequent gestations.

## Discussion

This primigravida lacked typical risk factors such as prior surgery, anomalies, or trauma, making spontaneous rupture in a primigravida woman particularly rare. The rupture occurring at the fundus, an area usually structurally strong, adds further rarity. The “sealed-off” nature of the rupture, wherein the herniated sac and fetus were localized within a peritoneal pocket, likely prevented major hemorrhage and maternal shock, masking the severity of the event and delaying diagnosis. Similar presentations have been reported by Hawkins et al. [3] and Verma [4], supporting the variability and subtlety of uterine rupture presentations, as also noted in recent case reports by Khan et al. [5] and Mishra and Mala [6].

This case contributes to rare reports of second-trimester rupture in primigravidas. Traditionally, uterine rupture is associated with factors such as a scarred uterus from previous cesarean section or uterine surgery, high parity, or excessive uterotonic use. However, this case highlights that primigravida patients without classic risk factors may also experience this life-threatening complication, particularly in the presence of undiagnosed uterine anomalies or spontaneous pathology.

The findings emphasize the importance of maintaining a high index of suspicion in any pregnant patient presenting with sudden abdominal pain, hemodynamic instability, or signs of fetal distress or demise, regardless of obstetric history. Delays in diagnosis can lead to catastrophic outcomes, including maternal hemorrhage, fetal loss, and even maternal mortality.

Therefore, rapid imaging-based assessment, especially with ultrasound and prompt surgical intervention, is critical in optimizing maternal outcomes. Early recognition and timely management, including uterine repair or hysterectomy when necessary, are essential to reduce morbidity. This case underscores the need for continued vigilance and clinical preparedness for atypical presentations of uterine rupture in all pregnant patients, not just those with identifiable risk factors.

## Conclusions

Second-trimester rupture in an unscarred primigravida uterus is rare but dangerous if not promptly diagnosed and treated. This case illustrates the importance of considering uterine rupture in differential diagnoses when pregnant patients present with abdominal pain and vaginal bleeding, even early in gestation and in low-risk individuals.

Radiological imaging, especially ultrasonography, plays an indispensable role in early diagnosis. The “sealed-off” nature of this rupture highlights a less common but critical variant that can delay clinical detection. Awareness and education about atypical presentations of uterine rupture are essential among all healthcare providers involved in prenatal and emergency obstetric care. Ongoing documentation and analysis of such rare cases are necessary to inform future guidelines and prevent maternal morbidity and mortality in similar clinical contexts.
